# Evoking the epistemology of climate governance through indigenous knowledge systems for sustainable development in rural Zimbabwe

**DOI:** 10.4102/jamba.v13i1.1024

**Published:** 2021-04-28

**Authors:** Shingirai S. Mugambiwa

**Affiliations:** 1 Department of Social Work, University of Limpopo, Sovenga, South Africa

**Keywords:** climate change, governance, indigenous knowledge systems, adaptation, resilience

## Abstract

This article seeks to establish the role of indigenous knowledge systems (IKS) in climate governance in pursuit of sustainable development in rural Zimbabwe. Rural communities in the developing world suffer the most from the negative effects of climate change. As such, their success in combating the effects of climate change is through establishing culture-specific methods. These methods constitute what I refer to in this article as climate governance through IKS. The impacts of climate change faced by rural communities include water shortages, drought, and floods, to mention a few. Drought is anticipated to bring about adverse consequences to water supply, which negatively affects food production and the environment in its entirety. Hence, this study investigates the methods of IKS water harvesting and other IKS-based adaptation and overall governance methods. The study employed a qualitative method in which participants took part in in-depth interviews and a focus group discussion (FGD) and data was analysed through thematic content analysis (TCA) and grounded theory. The study found that the role of spirit mediums and spirituality is essential in IKS climate governance. It also established that one of the most famous forms of climate change adaptation strategies in Mutoko district is IKS-based irrigation. The role of social networks was found to be essential in the sharing of ideas pertaining to irrigation and other adaptive methods of farming. Through the findings of the study, I developed a model that reflects and interprets indigenous-based climate governance structure in Mutoko district.

## Introduction

Rural communities in the developing world suffer the most from the negative effects of climate change. Bhusal (2009) asserts that rural communities in Africa engage one another in order to share their experiences of climatic conditions, ecosystem function and biological systems. This article explores the various levels of climate governance that exist in a Zimbabwean rural community. These levels include individual, village, political and development agencies that include non-governmental organisations (NGOs). Based on the findings of the study, a model of indigenous knowledge systems (IKS) climate governance was developed. The model is a unique overview of the indigenous climate governance that exists in African communities. The emphasis in this trajectory is on the significant role of traditional leaders and spirit mediums who are indigenous religious specialists and at the centre of the model are community members who are a common denominator in the IKS climate governance structure. To put this into perspective, other organs within the model such as NGOs and government, Rural District Council and smallholder farmers, in general, all work together to ensure that the community as a whole achieves sustainable development and other outcomes of a comprehensive climate change adaptation and resilience system. As such, the main objective of this article was to provide a theoretical understanding of climate governance from an African indigenous perspective. In order to achieve this objective, the model that was developed is entrenched in the premise that African epistemology is rooted in African ontology and the epistemological view of the traditional African is consonant with his or hermetaphysics. This implies that the knowledge of climate governance and practices around it in an African context cannot be separated from the African way of life vis-à-vis IKS. Based on the findings of the study the article presents a conceptual model that reflects and interprets the indigenous-based climate governance structure in Mutoko.

### Problem statement

Climate change has been established to be a global phenomenon that has severe effects on the livelihoods of communities and is expected to affect all regions and countries in some way (IPCC 2007). Rural communities in sub-Saharan Africa are faced with a plethora of socio-political, cultural and economic challenges that are a result of climate change (Nkoana et al. 2018). Climate change is expected to pose serious effects on the livelihoods of communities and some of the anticipated impacts include increased incidence of drought and floods, which result from erratic rainfall patterns and consequently have severe impacts on small-scale farming (IPCC 2007, 2013; Mugambiwa & Tirivangasi 2017). Many communities in the developing world significantly rely on the natural environment which entails natural rain for their crops, wild fruits and vegetables for their meals and wild vegetation and water for their domestic animals. Therefore, small-scale farmers are usually left with limited ability to cope up with escalating destructive disasters and this coincidentally affects rural livelihoods and food security. With the use of IKS, local communities play an important role in shaping adaptive capacity targeting the most vulnerable small-scale farmers.

### Study objectives

The study objectives were:

- To establish the role of IKS in climate governance in pursuit of sustainable development
- To develop a conceptual model that explains climate governance from an IKS perspective

### Research question

What is the role of IKS in climate governance?

## Literature review

### Climate governance

The generic understanding of the concept of governance refers to the management of resources and policy-making by means of exercising authority (Preti 2004). As such, the term governance is often used in order to signify a complex set of structures at both public and private levels and it is commonly associated with national administration. Rao (2008) defined governance as the exercise of political and administrative authority at all levels in the process of managing a country’s affairs. The definitions of governance provided in this article suggest that governance is a concept that deals with all processes that are related to the overall management of either an institution or a country. The concept of IKS plays a significant role in climate governance in rural communities (McGregor 2004). Indigenous knowledge systems is context-specific implying that every community in either developed or developing and underdeveloped countries has their own form of IKS (Nyong, Adesina & Osman 2007). That, therefore, triggers the quest to understand what IKS is and what it essentially entails. It has been documented that indigenous people who live in the vicinity of natural resources observe the activities around them and they identify and adapt to any changes in different ways (Mugambiwa 2018; Nhemachena 2007).

### Understanding epistemology and ontology

Smith (2003:1) defined ontology as ‘… a branch of philosophy which is the science of what is, of the kinds and structures of objects, properties, events, processes and relations in every area of reality’. Moreover, Tennis (2008:103) defined epistemology as ‘how we know and how we make implicit epistemic statements about knowledge of concepts, acts (such as representation), entities and systems’. In doing so, we create knowledge and our epistemic stance dictates what kind of knowledge that is. The understanding of the African culture and conceptions of reality leads to an improved understanding of the African approach to knowledge. The centrality of traditional African thought is born out of the premise that there exist ancestral spirits whose intentions are known to African people. Fontein (2006) suggested that spirit mediums (*Masvikiro*) command spiritual authority despite the fact that they do not receive recognition in local and national state structure. Fontein further contended that spirit mediums are not ancestors themselves but representatives of the ancestors who guide the living.

## Theoretical framework

### Grounded theory

**FIGURE 1:**
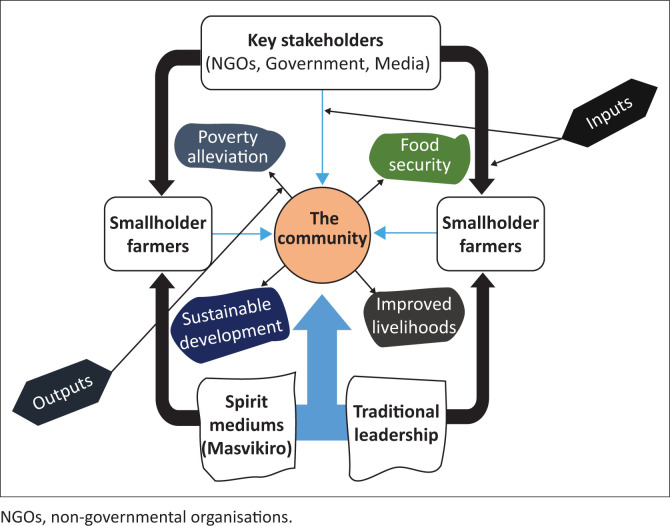
Climate governance through the indigenous knowledge systems model.

Grounded theory was propounded by sociologists Barney Glaser and Anselm Strauss. The creation of the theory was informed by the high levels of displeasure Glaser and Strauss had with the existing theories that dominated sociological research (Glaser 1992). The fundamental argument was that there is a need for researchers to be able to formulate theories from the findings of social research rather than rely entirely on the existing theoretical perspectives. Furthermore, Glaser (1992) asserted that grounded theory should be employed as a general methodology of analysis that is closely associated with data collection that makes use of the data to formulate a theory using systematically applied set of methods. Glaser and Strauss (1967) opined that the other importance of grounded theory is to help forestall the opportunistic use of theories that hold a dubious fit. Their argument was that there is a tendency amongst researchers to produce highly empirical research whose conclusion has a tacked-on explanation borrowed from a logically deduced theory. Glaser and Strauss (1967) presented what is termed logico-deductive theorising. This is how the sociologist makes use of selective examples systematically and allows them to have theoretical control over his formulations. Grounded theory entertains the theoretical saturation that evolves from data collection. As such, following the dictates of grounded theory, I developed a conceptual model of IKS climate governance (see Figure 1). The other reason for the adoption of grounded theory was that the concept of IKS climate governance is seemingly new and situating it within existing theoretical paradigms was problematic and limiting.

## Research methodology

### Study area

**FIGURE 2:**
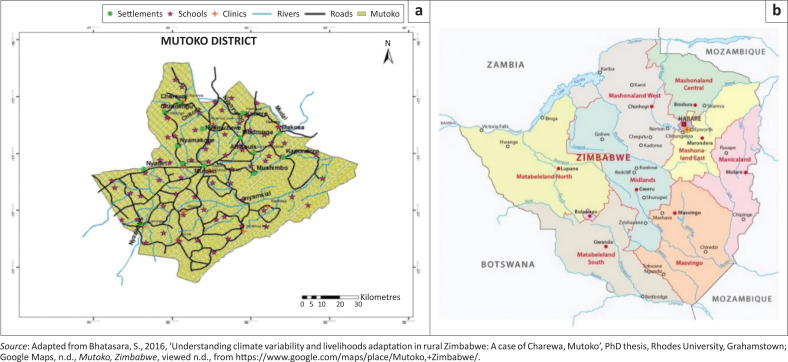
(a) Map of the Mutoko district, (b) Mutoko, Zimbabwe.

Mutoko district (see Figure 2) lies within Mashonaland East province (MEP) of Zimbabwe. The district covers 4092.5 km^2^ (Mvumi, Donaldson & Mhunduru 1988). The 2012 population census revealed that the district has 146 127 people (Moyo 2016). Mutoko was established as an administrative station in 1911 and it lies within 143 km from Harare, the capital city of Zimbabwe. The district was named after the local Chief Mutoko and the area is occupied by the *Buja* people. The district consists of a growth point, communal areas, resettlement farms and small-scale commercial farms. The growth point is populated at 198 people per km^2^, whilst the communal areas are at 46, resettlement farms at 23 and small-scale commercial farms at 10 (Bhatasara 2016).

### Methods

This research employed a cross-sectional and exploratory design. The population of the study comprised all smallholder farmers and institutions that are involved in climate change. Criterion purposive sampling was employed to select research participants from the villages in the district. Thirteen participants were selected for in-depth interviews (Table 1), 10 for a focus group discussion (FGD) (Table 2) and two key informants (KIs) from different government departments, namely, Department of agriculture and the Cotton Company of Zimbabwe. Hence, the total number of participants who took part in the study were 25. The 13 participants who took part in in-depth interviews comprised adult residents of the community whose daily work activities are directly affected by the effects of climate change because all of them were smallholder farmers and that applies to the FGD as well. Data collection was informed by factors such as age of the respondents, period of stay in the area and general understanding of climate change, climate change adaptation programmes and indigenous practices used to adapt to the effects of climate change. Household in-depth interviews were conducted at the participants’ homesteads and the two KIs interviews were conducted at a cotton depot in one of the villages. For all the interviews both in-depth and FGD a tape recorder was used to capture the responses. The FGD took 80 min and the in-depth interviews took approximately 65 min each.

**TABLE 1:** In-depth interview participants.

Participant†	Age	Gender	Period of stay in Mutoko district
Jacob	88	Male	Since birth
Tilda	N/A	Female	32 years
Melania	N/A	Female	Since birth
Richmond	52	Male	Since birth
Derick	56	Male	20 years
Peter	N/A	Male	Since birth
Jeremiah	60	Male	26 years
Melody	75	Female	36 years
Stella	72	Female	Since birth
Paul	53	Male	Since birth
Henry	49	Male	Since birth
Richard	66	Male	Since birth
Eliot	47	Male	Since birth

† , Pseudonym’s were used for the participants.

N/A, not applicable.

**TABLE 2:** Focus group discussion participants.

Participant name†	Age	Gender	Occupation
1. Chihera	62	Female	Smallholder farmer
2. Muchenje	54	Male	Smallholder farmer
3. Nyashanu	39	Male	Smallholder farmer
4. Melody	75	Female	Smallholder farmer
5. Stella	72	Female	Smallholder farmer
6. Jacob	88	Male	Smallholder farmer
7. Charles	49	Male	Smallholder farmer
8. Mujuru	35	Male	Smallholder farmer
9. Hwende	37	Male	Smallholder farmer
10. Munemo	51	Male	Smallholder farmer

† , Pseudonym’s were used for the participants.

Data were analysed through thematic content analysis (TCA) and grounded theory was further employed in order to develop a conceptual model. Pseudo names were given to participants in order to protect their identity. I identified trends and patterns that developed from the data collected and then coded and classified them into different categories that were used to analyse climate governance and the use of IKS for sustainable development in Mutoko district. Braun and Clarke (2006) suggested steps that should be considered in TCA. These steps comprise five phases that are discussed in the following sections.

#### Phase 1: Familiarising with the data

Familiarising oneself with data is the initial stage, which involves a careful reading of transcripts numerous times. In accordance with this stage, I went through all texts in the data by listening to the recorded texts more than once. This was important because it helped me to sift through the data and pick up all the relevant themes and aspects that were worth noting.

#### Phase 2: Generating initial codes

Generating initial codes is the second stage whose aim is identifying trends and patterns that would have developed from the data. After listening to the recordings repeatedly, I picked up the trends and patterns thereof and coded and classified them into different categories.

#### Phase 3: Searching and reviewing themes

The third phase comprises the searching and reviewing of themes, which conversely took a top-down approach where I used readily-made categories and identified instances fitting into those categories. The aim was to formulate concise phrases that interpreted the use of IKS in climate governance and together with the use of grounded theory I was able to develop the conceptual model.

#### Phase 4: Searching, defining and naming themes

The third stage was the searching, defining and naming of themes, which consisted of transforming notes into possible emerging themes. In this phase, I identified common themes that emerged from the data. This was done following the procedure recommended by Braun and Clarke (2006) in which the researcher looked for connections between emerging themes and grouped them together in accordance with their conceptual similarities.

#### Phase 5: Interpreting and compiling information

Phase five which is the final step enabled me to compile interpretations in a written account. This stage involved the interpretation and analysis of the data, which was guided by the dictates of grounded theory, Afrocentricity and Sustainable Livelihoods Approach (SLA) to oversee the development of a conceptual model for IKS climate governance. Accordingly, data from FGD and in-depth interviews was analysed using the same method. Hence, the two analysis methods, TCA and grounded theory were used interchangeably in order to comprehensively achieve the overall objectives of the study.

Following the tenets of grounded theory, the first step was to code the data collected accordingly, which was conducted following the dictates of both TCA and grounded theory. The coding was conducted through consistent observation of the trends and themes that emerged from the study which later played an important role in the theoretical formulation which is termed theoretical saturation in grounded theory terms. Strauss and Corbin (1990:56) asserted that ‘… categories designate the grouping together of instances such as events, processes, occurrences that have common central features with one another’.

## Findings and discussion

### Demographic data of participates

#### Major themes

Spirit mediums and spirituality: A FGD with 10 community members (Table 2) was conducted and amongst them was a headman of one of the villages. One of the topics discussed was about the role of spirit mediums in climate governance (Table 3). It emerged from the discussion that the role of spirit mediums and spirituality is essential in IKS climate governance. It is important to note that African people are spiritual and their spirituality is largely connected to spirit mediums (Fontein 2006). From the discussion, it emerged that spirit mediums known as *Mhondoro* in the *shona* language have control over the natural world. Rains, rivers and dams are directly influenced by spirit mediums. Also, mineral resources in the area are influenced by spirit mediums. If there are not enough rains in a particular season, smallholder farmers receive little farm produce, spirit mediums open mines and there will be abundant mineral resources such as gold. Furthermore, the inquiry into the role of spirit mediums discovered that the communities in Mutoko have great regard for spirit mediums. The function of spirit mediums is perceived as guardian angels who oversee communities in their day-to-day lives. Hence, it was found that when communities embark on any developmental projects such as the construction of dams and building of bridges, they consult spirit mediums first and failure to do so would result in the collapse of the dam before completion. One of the participants had this to say:

‘… We consult spirit mediums in many cases because we believe they look after us and they know what we want at any given point. I want to give you an example, the rains which resulted in cyclone Idai that we recently experienced came as a result of consultations with the ancestors because we had experienced successive dry spells …’ [Male, farmer, 35 years old, Focus Group Discussion July 2019]

**TABLE 3:** Major themes.

Theme	Explanation
1. Spirit mediums and spirituality	It emerged from the discussion that the role of spirit mediums and spirituality is essential in IKS climate governance.
2. Indigenous knowledge systems based irrigation	One of the most famous forms of climate change adaptation strategies in Mutoko district is IKS-based irrigation
3. Indigenous knowledge systems-scientific knowledge nexus in irrigation systems	It emerged that there is an interplay between Scientific and indigenous methods used for irrigation.
4. Indigenous management of resources	It emerged that indigenous management of resources is one of the ways in which the community deal with climate based and other challenges over the years.

IKS, Indigenous knowledge systems.

From the narratives of the participants, this article reveals that spirit mediums and spirituality play a very important role in climate-related issues vis-à-vis the development of communities in Mutoko district. The phenomenon of climate change and variability seem to be highly related to the existence of spirit mediums and their anger is also highly connected to preceding weather-related calamities. In support of this finding, Jiri, Mafongoya and Chivenge (2015) asserted that in Southern Africa, spiritual rainmaking ceremonies are at the heart of many traditional societies such as Mutoko. Moreover, Jiri et al. (2015) revealed that these rituals are performed by conducting prayers, using medicine portions, brewing and drinking traditional beer and dancing under trees amongst other activities as a way of manipulating the falling of rain. The activities are considered effective in yielding positive results amongst African indigenous people.

### Indigenous knowledge systems-based irrigation

One of the most famous forms of climate change adaptation strategies in Mutoko district is IKS-based irrigation (Table 3). Lack of sufficient rains that has become a common phenomenon over the years in Mutoko and many other places has seen irrigation becoming an effective adaptation strategy. There are many forms of irrigation that are practised in the area. To better comprehend this, one of the participants had this to say:

‘… the rivers have dried prematurely and it is very worrying because we depend heavily on them. It is part of our indigenous agricultural system that when the rains go we use water from riverside wells to irrigate our plants …’ [Female, farmer, 62 years old, Focus group discussion July 2019]

River waterholes seem to be playing an important role in this form of irrigation. However, another challenge that the community is facing relates to the early drying of rivers and dams. One of the participants indicated:

‘… The local rivers such as Nyamuzizi River have dried so early. In previous years the river used to go up to October with water flowing. However, due to early drying of the river, we now rely on the use of wells and boreholes. We have inspired one another to divert to the use of this system of irrigation.’ [Male, framer, 54 years old, Focus group discussion July 2019]

During field work, I encountered five smallholder farmers who were using this method in three different communities, namely Nyamuzizi, Matedza and Chibeta village. It emerged that a farmer in Matedza who had successfully employed the system was inspired by another farmer in Nyamuzizi village who had also successfully employed the system. In support of this finding, Lemma (2016) emphasised the importance of copying and adopting skills from others as an important strategy of adaptation. The author termed the process as ongoing learning, analysis, planning and adjustment, which should be employed in order to adapt to current and future disasters.

### Solar-powered borehole irrigation

**FIGURE 3:**
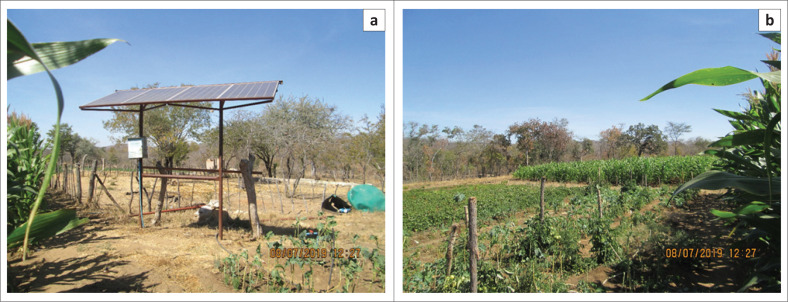
(a and b) A submersible solar-powered borehole irrigation system in Matedza.

**FIGURE 4:**
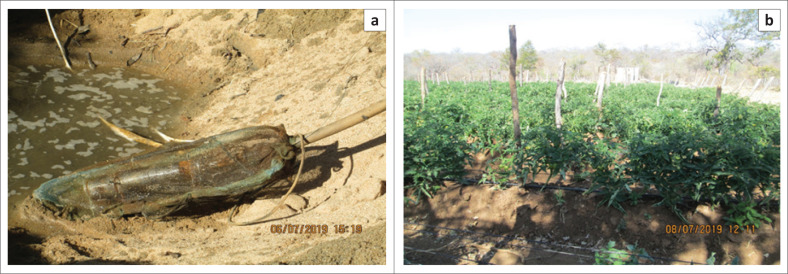
(a and b) A submersible solar-powered river waterhole irrigation system in Matedza village.

Solar powered borehole irrigation systems are widely used by local farmers as a form of climate change adaptation. Figure 3 shows a submersible solar-powered borehole used to syphon water for irrigation purposes. This is one of the most effective ways, which is used by a few smallholder farmers. Only a few farmers are using the system because it is expensive. The farmer in images in Figure 3 revealed that he rotates various crops throughout the year. These crops include potatoes, maize, beans, tomatoes and onions. Another system that was revealed in the study is whereby farmers draw water from river waterholes. The system is much cheaper compared with the borehole system. Images in Figure 4, demonstrates one such system in Matedza. I encountered two farmers who were using the system and they acknowledged that the system is effective and every serious farmer ought to employ it.

Moreover, the way the use of the system is spreading widely is a result of social networking amongst farmers who are adopting the system in the area. Ellis and Bahiigwa (2003) supported the use of social networks in climate adaptation as instrumental. The argument was that social networks are responsible for increasing awareness and use of adaptation options. This is what social scientists refer to as social capital, which is highly accredited to be a public good meant to enhance the exchange of resources and information amongst individuals. Similarly, Adger (2012) is of the opinion that social capital is a fundamental asset that is used to build a comprehensive climate change adaptive capacity. This form of networking is not facilitated by governments or other authoritative bodies but by ordinary community members who are involved in the day-to-day farming activities. The form of engagement carried out by the farmers can be interpreted as self-organised facilitation that is more sustainable and effective compared with those with adaptation mechanisms imposed by external entities.

Figure 4 demonstrates a submersible solar-powered river waterhole irrigation system in Matedza. The farmer makes use of the river waterhole that he keeps digging off to harvest water for irrigation purposes. The system requires the farmer to insert a submersible solar pump in the river waterhole or hand-dug well in order to syphon water. When the water levels decrease in the river waterhole, the farmer digs off much deeper to reach sufficient water levels. The process is an effective adaptation strategy employed by the farmer because this is done when the rains are long gone and the river has dried. This is usually a time wherein most community farming activities would have ceased completely whilst farmers are preparing for the next rain season in order to resume farming.

### Indigenous management of resources

**FIGURE 5:**
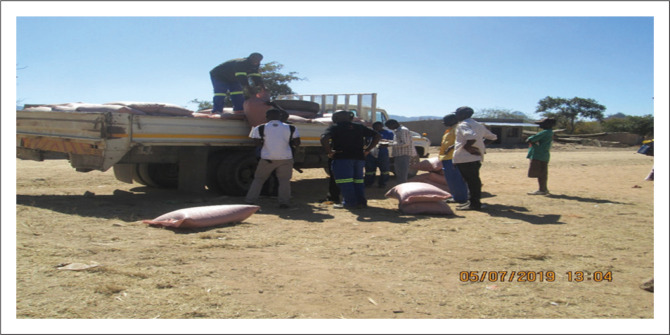
Villagers receiving government subsidised maize in Chibeta village.

Indigenous management of resources has always been one of the ways in which Africans deal with climate based and other challenges over the years. This study revealed that there is a relief programme that is administered at the village or ward level where the village headman is responsible for resource management and distribution in his village (Figure 5). This research has indicated that the village headman possesses records of people in his area of control such that when there are resources that the village ought to benefit from, he puts together the names of the community members and if there is a subsidised payment he also collects the payment on behalf of the supplier. Farmers in Mutoko communities have often received agricultural inputs from the government and other contractors such as Southern cotton and Cotton Company of Zimbabwe (COTCCO). In the event that there are such resources that need to be distributed, the village headman is responsible for the distribution of government inputs amongst many other roles. This form of indigenous management of resources significantly borrows from an ancient Shona practice known as *Zunde raMambo* (the chief’s granary), which is explained later in this article.

Indigenous management of resources where traditional leaders are fundamental in the distribution of resources such as government inputs is a fundamental indigenous aspect that relates to ancient practices in Shona communities. Traditional leaders played a key role in all aspects relating to the well-being of the community. For instance, the Shona culture has what was known as *Zunde raMambo* (the chief’s granary). Bhatasara (2016) described *Zunde raMambo* as a strategy that was used to address issues pertaining to food insecurity that people in the area have always employed at the community level over the years. It is an indigenous food security strategy where households contribute various grains such as maize, sorghum, rapoko and millet to *Zunde raMambo*. The grain would then be given to deserving households at the chief’s discretion in cases of droughts and other emergent crises. In this study local headmen revealed that one of their responsibilities was to provide government inputs to farmers and offer guidance on how to execute agricultural activities. For instance, one of the local headmen had a manual for cotton farmers that he used to provide guidance on appropriate farming methods.

**FIGURE 6:**
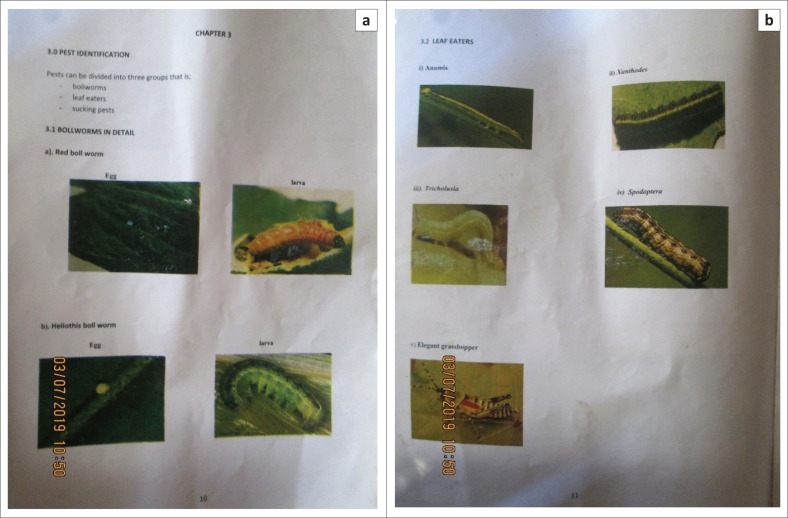
(a and b) Extracts from the headman’s manual for cotton farmers.

Figure 6 shows extracts that were captured from a village headman’s manual, which he used to help smallholder farmers to manage their crops. In the manual, there are images that identify the types of pests prone to the cotton crop, types of soil and fertilisers required. This was found to be helpful because the farmers improve their harvests by being aware of the circumstances around the crop. The headman’s role in this regard significantly complements the role of Department of Agricultural, Technical and Extension Services (AGRITEX) officers who work in the area to help farmers with initiatives aimed at improving their farming activities. The role of the headman in this regard is also of paramount importance in indigenous resource management. The knowledge from the manual helps the village headman to assist the farmers to produce good harvests because they would have succeeded in overcoming pests and selecting good soil and fertilisers for the cotton crop.

## Climate governance model

### An introspection of the model

The model (Figure 1) reflects and interprets the indigenous-based climate governance structure in Mutoko. The term IKS consequentially provokes one to imagine an ancient way of doing things. However, this study undressed the hidden meaning behind the term. Numerous definitions of IKS presented earlier amounts to the understandings, skills and philosophies that are engineered by communities bearing in mind the histories of interaction with their natural surroundings. The various organs represented in the model provide various services considered to be inputs meant to assist the community to adapt to the effects of climate change. The inputs are directed at the community and smallholder farmers. The community plays an important role in the structure of the model because the inputs from all organs are directed at the community and even though smallholder farmers are also an important recipient of inputs from all other organs, they refine and channel the inputs to the community in the form of farm produce. The nature of inputs directed to the smallholder farmers includes farming skills, agricultural inputs and irrigation strategies, which equips them to produce good harvests. The inputs directed to the community are multifaceted, that is, all organs involved are focusing on the community. The aim is to ensure that the community delivers sustainable outputs in the name of sustainable development. The model suggests that the ultimate goal of climate governance is to achieve four important outcomes referred to in this article as outputs. These are namely, poverty alleviation, food security improved livelihoods and sustainable development.

According to the model, the community forms the core of climate governance. All institutions represented in the model are aimed at the well-being of the community. For instance, key stakeholders such as the political leadership in rural communities that comprise councillors in every ward are there to deliver political mandate for the well-being of the community. The inputs in the model are aimed at enhancing the lives of the community and the outputs are the positive and successful fruition of the inputs vis-à-vis sustainable development in communities. As such the specific inputs and outputs are as follows:

#### Inputs

**Smallholder farmers:** Smallholder farmers are the backbone of all rural communities insofar as sustainable development is concerned. The role of smallholder farmers is to provide food for the community. They determine the state of food security in their communities. However, they are directly affected by the effects of climate change. They need to be equipped at all times so that they have sustainable adaptation strategies in place. The manner in which smallholder farmers adapt to the effects of climate change determines the state of sustainability in any community.

**Key stakeholders:** Amongst key stakeholders, there are government agents such as the media and NGOs. The role of these institutions in climate governance is fundamental. Some of these institutions work hand in hand with the government yet others are independent but at the end of the day the focus is to improve the livelihoods of the communities in the face of climate change. The mass media is a very important institution in climate governance. The findings of this study revealed that smallholder farmer and community members, in general, rely on the mass media for updates on weather forecasting by the meteorological department.

**Traditional leadership:** Traditional leadership is key to climate governance. One of the most important roles of traditional leadership is to ensure that the community observes their traditional rules and values concerning the management of natural resources and climate in general. Traditional leaders are the custodians of customary law and scholars such as Mohamed-Katerere (2002) and Turner and Clifton (2009) emphasised the importance of traditional leadership in climate governance and opined that recognising traditional leadership enhances the environment–spiritual connection, which is fundamental in the regulation of resources and the productivity of the farmers. In the model, traditional leadership is considered to be an essential organ that renders inputs to both farmers and the community at large.

The elders (***Vakweguru***) and Spirit mediums (***Masvikiro*):** The elders are all elderly people in the community. They play a fundamental role on issues pertaining to indigenous practices and customary law. They are one of the major channels through which IKS is passed because they pass the wisdom and knowledge from one generation to another. Spirit mediums are immensely important in climate governance. There are numerous cultural practices that are observed in African communities. These include rain making ceremonies (*Mafuwe*) and other rituals which cannot be executed without the instruction of spirit mediums known as *Masvikiro* since they are the ones who have access to God and the ancestors.

#### Outputs

**Poverty alleviation:** Poverty alleviation was presented as one of the outputs in the model. One of the major effects of climate change in rural communities is poverty. The effects of climate change hinder agricultural productivity, which results in widespread poverty and hunger amongst community members.

**Food security:** Food security is a fundamental aspect of the overall development of the community. Ahmed and Abah (2014) suggested that food security exists when communities at a family level have access to food in quantity and quality that is considered to be adequate and consistent all the time. The effects of climate change negatively affect the productivity of farmers, which results in challenges pertaining to food insecurity. Climate change is associated with various weather calamities, which include hot temperatures, lack of sufficient rains and erratic rainfall patterns. As such, the inputs reflected in the model are aimed at ensuring that smallholder farmers achieve their full potential so as to acquire one of the major outputs, which is food security.

**Improved livelihoods:** The livelihoods of the community are of paramount importance in the overall deployment of any community. Climate change affects the livelihoods of communities in their entirety. The sustainable livelihoods approach reiterates that ‘… A livelihood comprises the capabilities the capabilities, assets (including both material and social resources) and activities required for a means of living’ (Chamber & Conway 1991:43). As such, the various inputs presented in the model are fundamental in achieving improved livelihoods as an output.

**Sustainable development:** Achieving sustainable development is one of the major objectives of climate change governance. This is so considering the fact that climate change poses serious threats to communities and their natural resources. Sustainable development is defined as the principle and efforts aimed at meeting human development aspirations whilst at the same time sustaining natural systems’ ability to provide the natural resources upon which the economy and society depend (Shaker 2015). Hence, in the IKS climate governance model developed in this study the key stakeholders, traditional leaders and spirit mediums play a significant role in the provision of support (inputs) to the community and smallholder farmers.

## Conclusion

Indigenous knowledge systems in climate governance have been presented as essential in rural communities because of their history, values and belief systems. This fundamental conclusion also emerged from the IKS climate governance model that I developed. The model presents a form of governance that is holistic, empowering and participatory in nature. This is because all major organs of the African indigenous society and the other supporting organs and their roles are all deemed essential. This is achieved by the understanding and cherishing of the African cultural and conceptions of reality, which allow all stakeholders to take part in processes relating to the overall development of the community. This is largely opposed to the ideological framework of European colonisation that hails the supremacy of Western reason over non-Western people and cultures. As a result, the distinctive manner in which the African world is perceived constitutes African epistemology, which largely informs the model established in this article. My submission is that rural communities identify with IKS but the use of IKS entirely is not sufficient to bring about anticipated results. Hence, IKS in the modern world would only be successful if combined with SK.
